# Disruption of Cesarean Scar with Uterovesical Space Hematocele Mimicking an Endometrioma

**DOI:** 10.1089/whr.2021.0152

**Published:** 2022-05-02

**Authors:** Sujata Datta, Ritisha Basu

**Affiliations:** ^1^ Department of Obstetrics and Gynaecology, Fortis Anandapur, Kolkata, India.; ^2^ Department of Obstetrics and Gynecology, North Eastern Indira Gandhi Regional Institute of Health and Medical Sciences, Shillong, India.

**Keywords:** cesarean scar defect, endometrioma, cesarean complication

## Abstract

Our case highlights an extreme form of cesarean scar defect with diagnostic and surgical challenges.

The unusual presentation of a large extrauterine-encapsulated collection of altered blood and hemosiderin behind the posterior bladder wall that communicated with the endometrial cavity, through a full thickness myometrial discontinuity, at the site of a previous cesarean section, mimicked an endometrioma in an unusual location. This case report not only highlights the diagnostic challenge involved in this case but also highlights the surgical steps involved in the laparoscopic management of this extreme end of the spectrum of cesarean scar defects. We have attached a video of the laparoscopic surgery with step-wise description to shed more light on the management of this rare complication.

## Case Report

A 36-year-old lady presented with continuous vaginal bleeding for 10 months, after resumption of menstrual cycles 10 months after her second cesarean section.

**FIG. 1.**
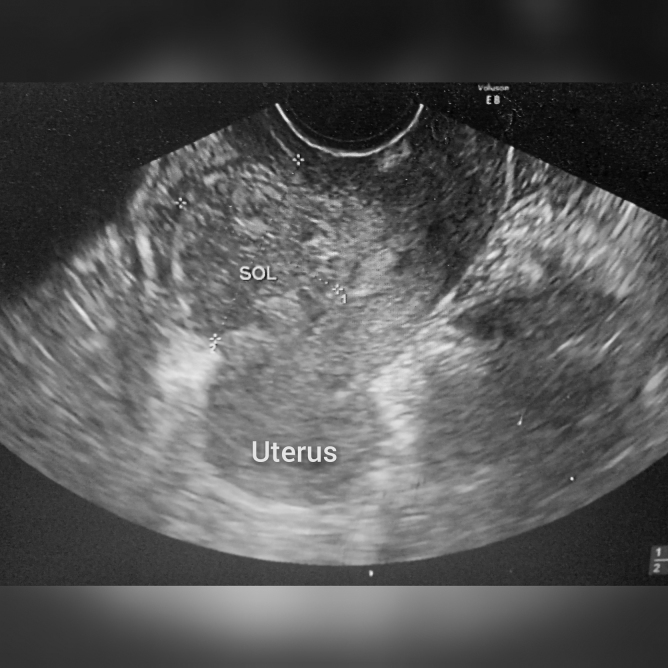
Transvaginal ultrasound image showing extrauterine heterogenous space occupying lesion communicating with the endometrial cavity.

A pelvic ultrasonography showed fluid in the mid-endometrial cavity communicating through the anterior lower uterine myometrium to a fluid containing extrauterine pouch, adjacent to the bladder (Fig. 1).

**FIG. 2.**
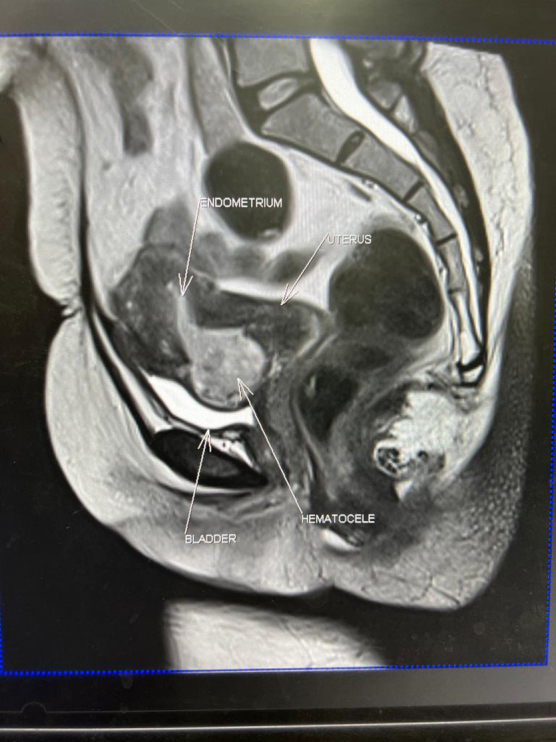
MRI image showing haematocele in caesarean scar site connecting to endometrial cavity. MRI, magnetic resonance imaging.

Magnetic resonance imaging showed a 1.5 cm cesarean scar defect, with a 3.1 × 2.8 cm lobulated heterogeneous lesion resembling an endometrioma, communicating through the anterior myometrial wall with the endometrial cavity (Fig. 2). The fat plane between the lesion and posterior aspect of the bladder was obliterated. A Ca 125 was 18.7 U/mL. A pregnancy test was negative.

She was treated with dienogest for 6 months. This suppressed the active bleeding. However, as there was no reduction in the size of the lesion, a hysterolaparoscopy was planned with a view to excising the lesion and repair the defect.

At hysteroscopy, the fundus and upper uterine cavity were found to be normal but a large defect in the anterior wall near the isthmus was seen while withdrawing the scope. Through this defect fat could be visualized.

Laparoscopy showed extensive omental, parietal, and bladder adhesions to the uterus, pulling it up to the anterior abdominal wall (Supplementary Video S1). These were dissected off after injection of diluted vasopressin into the myometrium to aid demarcation of the plane of dissection. The bladder and paravesical fat were dissected off the lower uterine segment to develop the uterovesical space. This revealed a thin walled pouch containing brownish fluid and hemosiderin deposit near the isthmus. The contents were cleared through suction and the sac lining was stripped off the posterior surface of the bladder and endometrial cavity.

The upper margin of the myometrial defect was refreshed with a harmonic scalpel to excise the fibrotic tissue. The defect was repaired with number 1 polyglactin absorbable suture in two layers, over a Hegar dilator placed through the cervix into the endometrial cavity to preserve canal continuity. Bladder integrity was confirmed with normal saline distension.

The patient was discharged on dienogest for a further 4 months after which she resumed normal menstrual cycles.

**FIG. 3.**
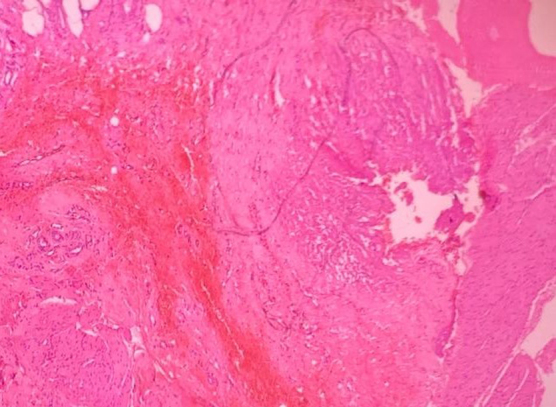
Histology slide showing haemorrhage and chronic inflammation.

Histology showed extensively congested fibrocollagenous stroma infiltrated focally by lymphoplasmacytic infiltrates (Fig. 3).

## Discussion

Our case is an example of one of the long-term complications of cesarean sections that is becoming increasingly common worldwide due to climbing cesarean section rates sometimes reaching up to 40%, in contravention to the recommended 15% rate suggested as optimal by World Health Organization (WHO).^1,2^

This unusual presentation of a large extrauterine-encapsulated collection of altered blood and hemosiderin behind the posterior bladder wall that communicated with the endometrial cavity, through a full thickness myometrial discontinuity, at the site of a previous cesarean section, mimicked an endometrioma in an unusual location. The rare presentation and atypical radiological findings posed a diagnostic challenge. The image characteristics though resembling an endometrioma did not fit with the classical sites of an endometrioma described in the literature. Moreover, no endometrial glands were found in the lining wall on histology that goes against the lesion being an endometrioma. Hence, it is best described as a hematocele in the uterovesical pouch communicating with the endometrial cavity through a cesarean scar defect.

At surgery, the encapsulated extrauterine lesion in the uterovesical pouch was exposed by adhesiolysis and the capsule lining the hematocele was stripped off. The myometrial defect was repaired to prevent further pooling of blood in the uterovesical space.

Unlike a typical cesarean scar niche or isthmocele, which is a partial defect in the healed cesarean scar, our patient had no measurable residual myometrial tissue surrounding the collection anteriorly, being lined instead by a wall of fibro collagenous tissue on histology. This confirmed a full thickness disruption of the cesarean scar, possibly due to poor healing of uterine incision.

A common symptom of the spectrum of cesarean scar defects is continuous postmenstrual bleeding (30%).^3^ The menstrual blood that collected in the saclike extrauterine pouch flowed back through the large full thickness myometrial defect into the endometrial cavity, presenting as vaginal bleeding through the rest of the month.

Medical management with dienogest, which to our knowledge has not been used before for this indication, suppressed the symptoms but did not cause resolution of the lesion.

Our operative finding demonstrating dense fibrotic adhesions between the parietal abdominal wall, bladder, and lower segment of uterus lends credence to the hypothesis that describes an upward retraction force from adhesions that counteracts and impedes cesarean scar healing as a cause of cesarean scar defect or a niche formation.^4^

It is not known whether the method of uterine cesarean, either single or double layered, influences the healing and predisposes to the scar defect. However, as there was no bleeding during the first 10 months after the cesarean section, the blood was certainly menstrual blood and not accumulated blood due to primary bleeding from an incompletely closed cesarean incision.

Abnormal healing of cesarean scars can lead to partial defects that predispose to cesarean scar ectopics. However, our case represents the far end of the same spectrum, as it involved a complete disruption of a cesarean scar during the process of healing, to form an encapsulated hematocele in the uterovesical pouch.

## Consent

Consent taken from patient for publication of case report.

## Supplementary Material

Supplemental data

## Abbreviations Used

MRI: magnetic resonance imaging
WHO: World Health Organization
